# Training as imagined? A critical realist analysis of Scotland’s internal medicine simulation programme

**DOI:** 10.1186/s41077-024-00299-y

**Published:** 2024-06-26

**Authors:** Joanne Kerins, Katherine Ralston, Suzanne Anderson Stirling, Nicholas Simpson, Victoria Ruth Tallentire

**Affiliations:** 1 Scottish Centre for Simulation and Clinical Human Factors, Forth Valley Royal Hospital, Larbert, FK5 4WR UK; 2 Medical Education Directorate, NHS Lothian, Edinburgh, UK; 3 NHS Education for Scotland, Glasgow, UK

**Keywords:** Training evaluation, Critical realism, Internal medicine, Immersive simulation, Simulation-based mastery learning

## Abstract

**Background:**

Evaluating the impact of simulation-based education (SBE) has prioritised demonstrating a causal link to improved patient outcomes. Recent calls herald a move away from looking for causation to understanding ‘what else happened’. Inspired by Shorrock’s varieties of human work from patient safety literature, this study draws on the concept of work-as-done versus work-as-imagined. Applying this to SBE recognises that some training impacts will be unexpected, and the realities of training will never be quite as imagined. This study takes a critical realist stance to explore the experience and consequences, intended and unintended, of the internal medicine training (IMT) simulation programme in Scotland, to better understand ‘training-as-done’.

**Methods:**

Critical realism accepts that there is a reality to uncover but acknowledges that our knowledge of reality is inevitably our construction and cannot be truly objective. The IMT simulation programme involves three courses over a 3-year period: a 3-day boot camp, a skills day and a 2-day registrar-ready course. Following ethical approval, interviews were conducted with trainees who had completed all courses, as well as faculty and stakeholders both immersed in and distant from course delivery. Interviews were audio-recorded, transcribed verbatim and analysed using critical realist analysis, influenced by Shorrock’s proxies for work-as-done.

**Results:**

Between July and December 2023, 24 interviews were conducted with ten trainees, eight faculty members and six stakeholders. Data described proxies for training-as-done within three broad categories: design, experience and impact. Proxies for training design included training-as-prescribed, training-as-desired and training-as-prioritised which compete to produce training-as-standardised. Experience included training-as-anticipated with pre-simulation anxiety and training-as-unintended with the valued opportunity for social comparison as well as a sense of identity and social cohesion. The impact reached beyond the individual trainee with faculty development and inspiration for other training ventures.

**Conclusion:**

Our findings highlight unintended consequences of SBE such as social comparison and feeling ‘valued as a trainee, valued as a person’. It sheds light on the fear of simulation, reinforcing the importance of psychological safety. A critical realist approach illuminated the ‘bigger picture’, revealing insights and underlying mechanisms that allow this study to present a new framework for conceptualising training evaluation.

## Background

I saw the crescent; You saw the whole of the moon. The Waterboys, 1985

Quality simulation-based education (SBE) requires robust training evaluation. With reflection being a founding principle of SBE [1], educators too must reflect on the impact of their training interventions. Given it is incumbent on leaders of simulation programmes to ensure educational impact, there has been a field-wide focus on demonstrating improved patient outcomes as a result of educational interventions [2] . More recently, there have been calls to move away from demonstrating causality as a marker of success [2] and towards capturing unintended consequences, i.e. ‘what else happened?’ [3]. We are also encouraged to embrace holistic evaluative practices, involving multiple stakeholders and occurring iteratively at various points throughout the life of a programme [4].

However, within these expectations, there remains a largely unarticulated tension. Training evaluation is often required to secure funding and/or to prove an intervention is worthy of the investment of people and time [5]. This inevitably introduces a bias towards demonstrating positive outcomes and leads to a tendency to ignore unintended consequences, particularly negative ones. There can be divergent priorities of stakeholders [6] and tensions between intentions: are we trying to prove or to improve [7, 8]? This is problematic because, as simulation educators, we have much to learn from others’ mistakes and challenges, but our orientation toward outcome-based evaluation negates this opportunity. It also fails to attend to potential hidden impacts of SBE which, as a community, we ought to strive to understand as fully as possible.

In addition to shortcomings in attending to unintended outcomes, there is a lack of theory-driven evaluation [9] that might enable a deeper understanding of intervention processes. Within SBE in particular, there has been a paucity of formal programme evaluation using a scholarly approach [3]. We must engage with theory to develop programme evaluation practices capable of capturing complexity [10, 11]; SBE can be a particularly complex social intervention, for example through its propensity to evoke emotion [12]. We aim to answer the call for more scholarly approaches to programme evaluation [13], particularly within SBE, with our study.

### Conceptual framework

In our conceptualisation of programme evaluation, we were inspired by literature from a related but distinct field. In the patient safety literature, Steven Shorrock describes the concept that ‘work-as-done’ is distinct from ‘work-as-imagined’ and there are many proxies to better understand the realities of clinical work [14, 15]. This concept recognises that in clinical practice, work is often not conducted as might be imagined by various stakeholders and understanding work-as-done is crucial to deepening understanding of patient safety. This stems from a Safety II perspective, the system’s ability to succeed under varying conditions. This perspective embraces and understands that performance will inevitably diverge from work-as-imagined [15]. A clearer appreciation of the realities of work-as-done is crucial to engendering impactful evaluation and interventions that are not based on fantasy or work-as-imagined. Shorrock also describes other proxies for work-as-done such as work-as-prescribed (e.g. protocols) and work-as-disclosed (e.g. healthcare staff documentation) [14]. Similar to the realities of clinical practice as described by Shorrock, we recognise within this work that while we can anticipate some training impacts and their mechanisms, others will be unanticipated, and training does not always occur as imagined by those who have designed and delivered it.

In striving to fully understand the impact of a complex SBE intervention, this study takes a critical realist stance. Critical realism accepts that there is a reality to uncover, but acknowledges that our knowledge of this reality is inevitably our construction and cannot be truly objective [16, 17]. Critical realism focusses on understanding the mechanisms that drive social reality and offers explanatory power [18] which is key in our improvement efforts for SBE. It ‘acknowledges the existence of an external social reality and the influence of that reality on human behaviour’ [19]. This is particularly important to understand in the realm of SBE, which often involves complex social interventions.

**Fig. 1:**
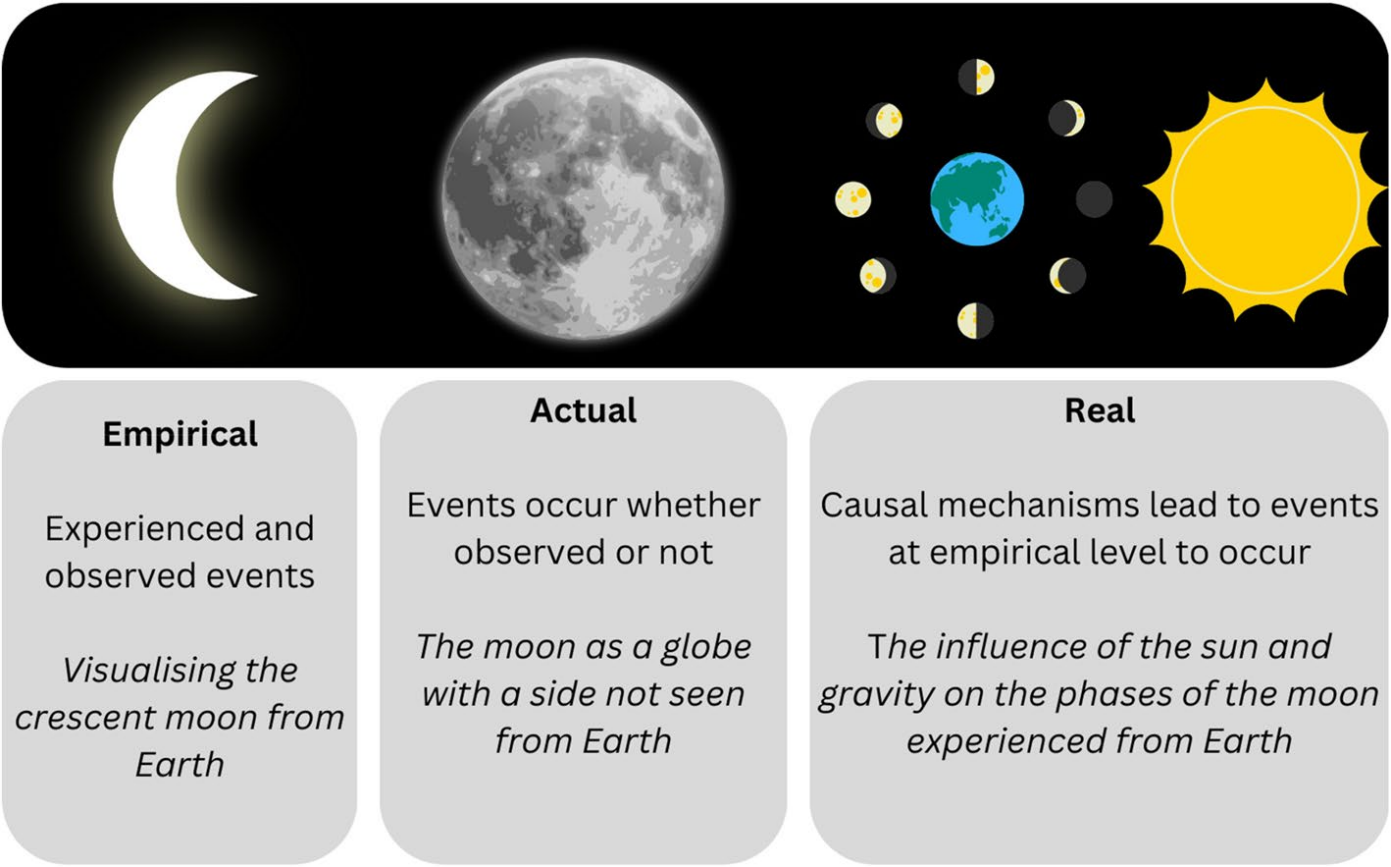
Critical realism levels of reality with illustrative example [20]

Critical realism considers three levels of reality as outlined in Fig. 1 with an illustrative example [20, 21]. Critical realism aims to find the best explanation of reality through engaging with theory and researcher-driver analysis [20]. This can be conceptualised by considering three levels of reality: empirical, actual and real [20].

Critical realism treats the ideas and meanings held by individuals as equally real as physical objects and seeks to explain and critique social conditions to produce claims for action on social problems [20]. By considering deeper levels of reality, beyond the descriptions of experienced or observed events, we can come closer to appreciating underlying mechanisms and hidden events [22]; closer to understanding the dark side of the moon. Integrating theory from another discipline, incorporated with a critical realist paradigm, aims to offer a nuanced approach to the questions of why, how and under what circumstances SBE works or does not work, in our context [23]. In doing so, we aim to unlock the black box of our national internal medicine training (IMT) simulation programme—the ifs and buts in the chain of causation [19, 24]. It is also noted that there is a lack of accessible material applying critical realist methodology [20], particularly in SBE, and this study presents and discusses how this methodology can be useful in the simulation context. Given Shorrock’s work-as-done is closest to reality with other proxies overlapping with this, this concept was chosen to inform our thinking about the simulation training intervention and how we can apply this theory to better understand training-as-done. In doing so, we metaphorically remove our rose-tinted glasses in an effort to ‘help us get closer to reality’ [20].

### Aim

This study aimed to understand the experience and impact, intended and unintended, of the IMT simulation programme in Scotland and the underlying causal mechanisms.

## Methods

### Philosophical orientation

The research paradigm for this study is critical realism. In terms of philosophical assumptions, this assumes that there is a reality to uncover (realist ontology) but acknowledges the subjectivity of our perspectives in doing so as researchers (subjective epistemology) [16, 25]. Critical realism developed as an alternative to positivism and constructivism, most notably through the work of Bhaskar [26] who advocated for the use of theory.

### Ethics

This study received ethical approval from NHS Education for Scotland (NES/Res/14/20/Med). Participants gave written consent for data collection and the publication of anonymised results.

### Study design

Bhaskar advocated for the use of theory as a starting point for empirical research [20, 26], without committing fully to the content of specific theories which can be advanced, modified or rejected during analysis. Our initial theory was informed by Shorrock’s proxies of human work [14], and so, in our context, the concept that ‘training-as-done’ will differ from ‘training-as-imagined’. Understanding and embracing this was the foundation of this study.

### Context

This study took place in Scotland and focused on the IMT Stage 1 Simulation Strategy, embedded within IMT in Scotland since 2019. IMT is a 3-year training programme for doctors in the UK pursuing a career in medical specialties once they have completed foundation training. The Joint Royal College of Physicians Training Board outlines the curriculum for this training programme which asserts a requirement for SBE [27]. The IMT curriculum is approved by the General Medical Council and trainees must meet curricular requirements each year of their training programme to progress to the next year [28].

**Fig. 2:**
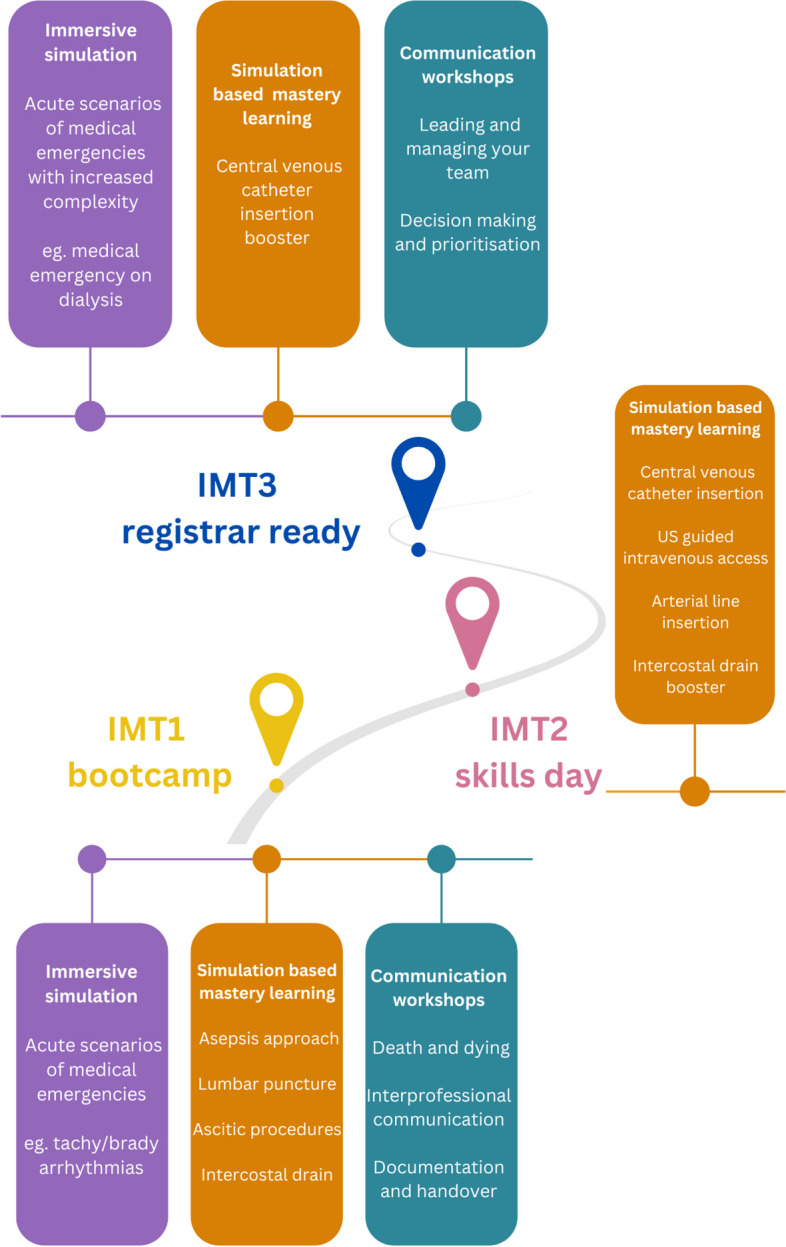
IMT Simulation Strategy in Scotland

In Scotland, the IMT simulation programme, delivered nationally, involves three courses over their 3-year training programme to provide simulation training targeting curricular requirements to support their training progression. This simulation programme constitutes a 3-day boot camp (year 1), a skills day (year 2) and a 2-day registrar-ready course (year 3). The courses incorporate a range of immersive simulation scenarios for training in acute medical conditions, simulation-based mastery learning (SBML) of procedural skills [29] and communication workshops involving simulated patients and tabletop simulation. A detailed description of the individual course components is displayed in Fig. 2.

To appreciate the scale of the simulation strategy, for the period including trainees involved in this study, the IMT1 boot camp was delivered to 122 trainees over seven 3-day courses in 2020/21; the IMT2 skills day was delivered to 135 trainees over seven 1-day courses in 2021/22; and the IMT3 registrar-ready course was delivered to 170 trainees over six 2-day courses in 2022/23. From a training delivery perspective, this is 40 days of simulation training delivered in each academic year. At the end of each academic year, a review day is hosted with key stakeholders including trainees, faculty, administrative staff and simulation technicians to reflect on feedback and consider improvements.

### Data collection and analysis

Data were gathered from trainees through pre- and post-course questionnaires at simulation training events across 3 years. Data were tracked for each trainee allowing, for example, trends of specific procedural confidence to be observed over their simulation training programme. This gave us *extensive* data at the empirical level [20]. This project aimed to gather *intensive* data through in-depth interviews with a range of perspectives to try to gain an appreciation of the actual and real levels of training-as-done as described in Fig. 1 [20].

After completion of all three simulation training courses, trainees were invited to participate in this research project via an online questionnaire. Consenting participants were contacted by email between 3 and 6 months after the completion of their final IMT simulation course and invited to an interview. Fifty-three trainees who consented were contacted via email to arrange an interview. After trainees responded and an interview date was arranged, individual trainee evaluation data across 3 years were reviewed with consent to prompt and discuss their experiences, with conversations guided by their feedback. Faculty attending the course’s annual review day in 2023 were invited to take part in the study to explore the gap between training-as-imagined and training-as-done. Both trainees and faculty consenting to interview were offered a range of potential dates for interview with convenience sampling of those able to attend. All interviews were conducted by JK, audio-recorded, transcribed verbatim and analysed, with the initial codes informed by Shorrock’s proxies for work-as-done [14].

Analysis was undertaken in parallel with data collection [18] which was a theory and researcher-driven process. Critical realist analysis involves an initial identification of demi-regularities, or tendencies in the data, at the empirical level which was led by JK forming initial codes for analysis based on early data collected. An example is a tendency for pre-simulation anticipation of trainees. Initial codes were developed using provisional proxies for training (training-as-imagined, training-as-prescribed, training-as-intended/unintended and training-as-transferred). Training-as-prescribed related to study participants’ perceptions of the IMT curriculum and its influence, rather than including analysis of the curriculum documentation itself. During this iterative process, we opted to seek perspectives from stakeholders who may or may not have had active involvement with course delivery, to understand training-as-imagined from an outsider perspective. Snowball sampling was utilised to identify stakeholders who could offer alternative perspectives with convenience sampling of those who replied to the interview invitation and were able to attend an interview from a range of offered dates. JK analysed each transcript, with each co-author analysing a subset of transcripts, including a range of trainee, faculty and stakeholder transcripts. NVivo (Version 12) was utilised for data management.

Codes changed iteratively on several occasions through discussions with the research team at regular intervals during data collection and analysis. In accordance with the critical realist analysis process of abduction, or theoretical redescription, codes were further developed with some initial codes being rejected and some added based on the data to develop the most accurate explanation of reality [20]. Further coding cycles by the lead author inductively identified organisational codes of design, experience and impact which provided an ongoing structure during analysis. During this coding cycle, a process of retroduction, which reasons why something is happening [30] and focusses on causal mechanisms at deeper levels of reality, was led by JK with discussion with all research team members [20]. Taking the earlier example of pre-simulation anticipation, an underlying mechanism highlighted through retroduction was that of peer observation.

### Reflexivity

The research team was purposefully compiled from a variety of perspectives. SAS is a specialist research lead at NHS Education for Scotland with extensive qualitative research experience, who is involved in the evaluation of IMT simulation but not in delivering the courses. JK is an acute medicine registrar with significant medical education research experience and involvement as faculty in the IMT simulation courses. VT is a consultant in acute medicine, a simulation educator and a post-doctoral medical education researcher and leads the IMT simulation strategy. KR is a geriatric medicine registrar with medical education research and simulation educator experience with minimal prior experience in the delivery of IMT simulation courses. NS has recently completed the IMT programme including all the IMT simulation courses and so has a unique perspective on data analysis from a trainee perspective. The research team met frequently during the data collection and analysis phases to discuss coding issues, refining codes iteratively with ongoing data collection and analysis. Given the tensions discussed around conducting evaluative work of a programme you have developed and continue to deliver, we purposefully included perspectives of those not involved in design (SAS, KR and NS) and a recent participant (NS). As a result, the conversations prompted reflection on the tensions between curriculum and clinical reality of workplace demands as well as the potential disparity between faculty perception of trainee experience and trainee reflection on experience. These discussions informed and influenced the resulting proxies and highlighted the benefits of multiple perspectives during evaluative work.

## Results

Between July and December 2023, 24 interviews were conducted including ten trainees, eight faculty members and six stakeholders. Two stakeholders had also been involved as faculty in training delivery but had additional leadership roles in IMT training which made them significant stakeholders in other ways. Interviews lasted an average of 43 min with individual averages of 40 min for trainee interviews, 55 min for faculty interviews and 34 min for stakeholder interviews. Participants are labelled T for trainee, F for faculty and S for stakeholder. The results are displayed in three main categories of design, experience and impact with proxies within each category, some of which aligned to the sensitising theory of proxies for human work and others that were inductively derived from the data. Within each category, we have summarised the proxies with a table including an illustrative example from our data.

### Design

In the design and planning phases of all three courses within the strategy, the course content was mapped to the IMT curriculum [28], with the curriculum being a prominent feature at design events. However, trainees were also invited to planning days and were able to voice their opinions on aspects they wanted to prioritise, based on felt needs in the clinical workplace. It was also apparent through faculty discussion that faculty clinical experience influenced their design and implementation, prioritising what they deemed most important for trainees:

You’re constantly reminded, you know, it’s got to be the curriculum, it’s got to be the curriculum. But I think, more than that, it’s not just been the curriculum. I think it’s been very much based on expertise of people on the ground…So, I don’t think we’re slaves to the curriculum, in that sense. (F1)

One of the challenges with a national course was that trainees possess a wide range of prior experience and their preferences varied.

I think it is good that it’s aligned to the curriculum, if you go on the basis that the curriculum is well designed for the purpose that is…But I think it’s nice also to not be so rigidly aligned and be a bit led by the trainees…and sim does that – it brings a lot of experiences so it does feel more trainee led than very like this is the specific area of the curriculum we’re following today. (F6)

Similarly, faculty were varied in their clinical specialty and tended to prioritise areas that are prominent in their daily practice. This could be conceptualised as a tension between these factors: three forces (curriculum, trainees and faculty), at times competing, with the resulting design a product of the ways in which these tensions interacted.

Study participants also reflected on the benefits of the way in which the courses are nationalised and therefore trainees receive simulation training-as-standardised:

I think IMTs in general didn’t get a lot of structured training. So I think there’s a strategy, it’s really nice having…like everyone gets that …At least everyone’s getting those kind of standards, which is nice, because I think otherwise IMT can be quite varied in other ways. (F6)

This was an intentional design feature of the national programme which yielded benefits for trainees, faculty and stakeholders such as training programme directors, with a shared understanding of the training that has been delivered to all. The proxies for training design from the data, as in Table 1, represent three main sources of influence in curriculum, trainees and faculty with a resulting standardised course designed for all.

**Table 1:** Proxies for training design

	**Proxy**	**Example**
**Design**	Training-as-prescribed	Need for central venous access training as described as included in the IMT curriculum
	Training-as-desired	Trainees keen for arterial line practice but not described as part of IMT curriculum by faculty
	Training-as-prioritised	Repetition of chest drain insertion due to faculty awareness of lack of opportunities in clinical practice
	Training-as-standardised	The same course format is delivered to all trainees regardless of prior experience

### Experience

The experience of the simulation training programme extended to pre-attendance anticipation as voiced by trainees. The experience itself involved both intended and unintended experiences, with significant overlap between these. The intended experience describes the planned experience through the intentional design of the courses, whereas the unintended experience represents the hidden experience, not considered at the design stage.

Training-as-anticipated was due to a ‘preconceived negative concept of sim’ (T8) which was apparent due to an expectation of performance, particularly under observation by peers. This anxiety is related to two aspects—immersive simulation scenarios of emergency situations and assessment of procedural skills on a mannequin.

I think every time I come across a course where I end up having to do sim, beforehand I say ‘I hate it’. I would always say it’s valuable, but I would never say beforehand it’s something I particularly enjoy to do. (T5)

This apprehension prompted pre-learning to be completed for the procedural skills; however, there was a sense of dread regarding the immersive simulation. This anticipation was nevertheless coupled with a sense of accomplishment and worthwhile experience following the session.

Some of this anticipation was intentionally dissipated by the creation of a psychologically safe learning environment and positive atmosphere through the ‘can do attitude’ (F8) of faculty. The simulation programme provided high-fidelity rehearsal, an intended experience, which was valued by trainees. In particular, the immersive simulation scenarios provided an opportunity for non-technical skills training. Through the process of attending and engaging with the course, trainees described the unintended experience of social comparison. They described ‘benchmarking’ (T5) themselves against their peers as a way of reassuring themselves that they are performing at the expected level for their training. This process is not readily available in the workplace where ‘you can be quite isolated’ (T6) with a lack of feedback, so the courses provided an opportunity to ‘feel like, yes, I’m at the kind of level that I’m supposed to be at.’ (T6).

Both faculty and trainees described how the simulation courses promoted a sense of identity among the IMTs, enhanced by a sense of social cohesion. This was a largely unintended overarching consequence by those involved in course design focussing on individual components. The advantage of ‘physically coming together’ (T7) prompted trainees to feel ‘part of a community’ (T7) ‘where they get to realise they are not alone’ (F8) but also allowed the opportunity for faculty to identify and offer support to trainees in difficulty. However, some noted that whilst the courses fostered a sense of community and identity, this was not always sustained when they ‘all go back to our regular jobs and that very rapidly dissipates.’ (T8) The national delivery also provided the unintended outcome of allowing trainees to discuss the similarities and differences between various hospitals and regions in Scotland.

So I think there’s a huge value in them getting a safe space to talk about their experiences and relate it, and that’s what sim does. (F6)

There was a sense from both faculty and trainees that having an awareness of this variety was beneficial for trainees to provide insights into alternative ways of working. The national delivery was intentional to provide consistency of training experience and access, but the opportunity during simulation to discuss and compare experiences was an unexpected benefit.

Training-as-imagined not only incorporates what faculty and trainees involved in the design process imagine the experience will be but also includes what those without first-hand experience of the courses think is taking place during such sessions. Most stakeholders had a good grasp of what the training courses entailed. However, some training programme directors were less aware of the programme content, which could curtail their ability to support the transfer of training to clinical practice.

It’s all very good talking about all the process and the concepts are brilliant, but it was still a bit pie in the sky when they’re talking about it all but I think it became crystal clear when you’re actually doing an aspect. (S4)

It was apparent that stakeholders with influence over providing facilities and ensuring trainees are released from clinical activities to attend were aware and convinced of the benefits of the courses for trainee development and wellbeing. This has been reinforced through the research and evaluation efforts ongoing throughout the training delivery.

The proxies for training experience are summarised in Table 2 with illustrative examples incorporating the way in which training is anticipated, how it is experienced in intentional and unintentional ways and how it can be imagined by various stakeholders.

**Table 2:** Proxies for training experience

	**Proxy**	**Example**
**Experience**	Training-as-anticipated	Anxiety relating to peer observation
	Training-as-intended	Opportunity for skill rehearsal
	Training-as-unintended	Social comparison to provide reassurance
	Training-as-imagined	Good feedback from trainees but lack of insight of training programme directors into aspects covered

### Impact

The impact of the simulation programme was evident across four areas. There were the intentional impacts of the transfer of training to practice for trainees, the somewhat unintended impact on faculty development, the ways in which the simulation programme inspired others and the ongoing impact of programme improvement—an iterative process of how training evolved and continues to evolve. Trainees found the simulated experience to be powerful in aiding their transfer of training to clinical practice. They valued the rehearsal in a simulated environment without ‘real-world stakes’ (T5) which they felt prepared them to ‘fall back on the same thought processes used for any urgent situation’ (T5). SBML for procedures was ‘empowering to take up opportunities’ (T7) after attending simulation courses.

Faculty were passionate about the benefits they had gained from being involved in course delivery.

I don’t recall any teaching session where somebody hadn’t brought something to the room that’s been interesting, or that’s thought provoking, or that I’ve learned something from. (F1)

Training delivery influenced the clinical practice of faculty in positive ways such as being ‘a much better proceduralist because that framework has very much become an entrenched part of my practice’ (F3). In addition to learning from and with trainees, they found networking with other faculty on a regular basis a beneficial process and a way of meeting other like-minded people. There was also a positive sense of contributing and ‘giving something back’ (F3). The sheer scale of the course in terms of the number of courses delivered with multiple cycles of individual sessions within each course led to repetition and ample opportunity for faculty development.

There were examples of how the IMT simulation courses had inspired others, including the ethos of having an over-arching strategy and vision that was seen as instrumental.

I’ve certainly taken the structural component from boot camp…and also taken the knowledge of how we teach (F8)

This included the ‘multimodal’ (S2) aspect with immersive simulation, SBML and communication workshops with ‘proper impact evaluation throughout it’ (S2). Given the number of times the courses run, and the amount of kit required, the courses had forced the team delivering training to become more organised in a way that was seen as beneficial.

The iterative way in which the programme is evaluated each year allows for constant re-assessment and changes to be implemented, which this work aims to support. For example,

The problem we used to have was that year 1 boot camp didn’t cover central lines for their requirements and now that’s been changed. (S3)

This leads to training-as-evolved, adapting to meet trainee needs and address issues as they are identified.

The proxies for training impact are summarised in Table 3 with illustrative examples including the impact for trainees, for trainers and for the course itself in terms of evolution and improvement. Training impact also included influencing others embarking upon similar ventures.

**Table 3:** Proxies for training impact

	Proxy	Example
Impact	Training-as-transferred	Performing procedures in clinical practice with the SBML approach
	Training-as-faculty development	High volume of courses allowing training of junior faculty in debriefing
	Training-as-inspiration	Spread of multimodal design to other projects
	Training-as-evolved	Incorporating central venous access into IMT1 boot camp in response to curricular changes

**Fig. 3:**
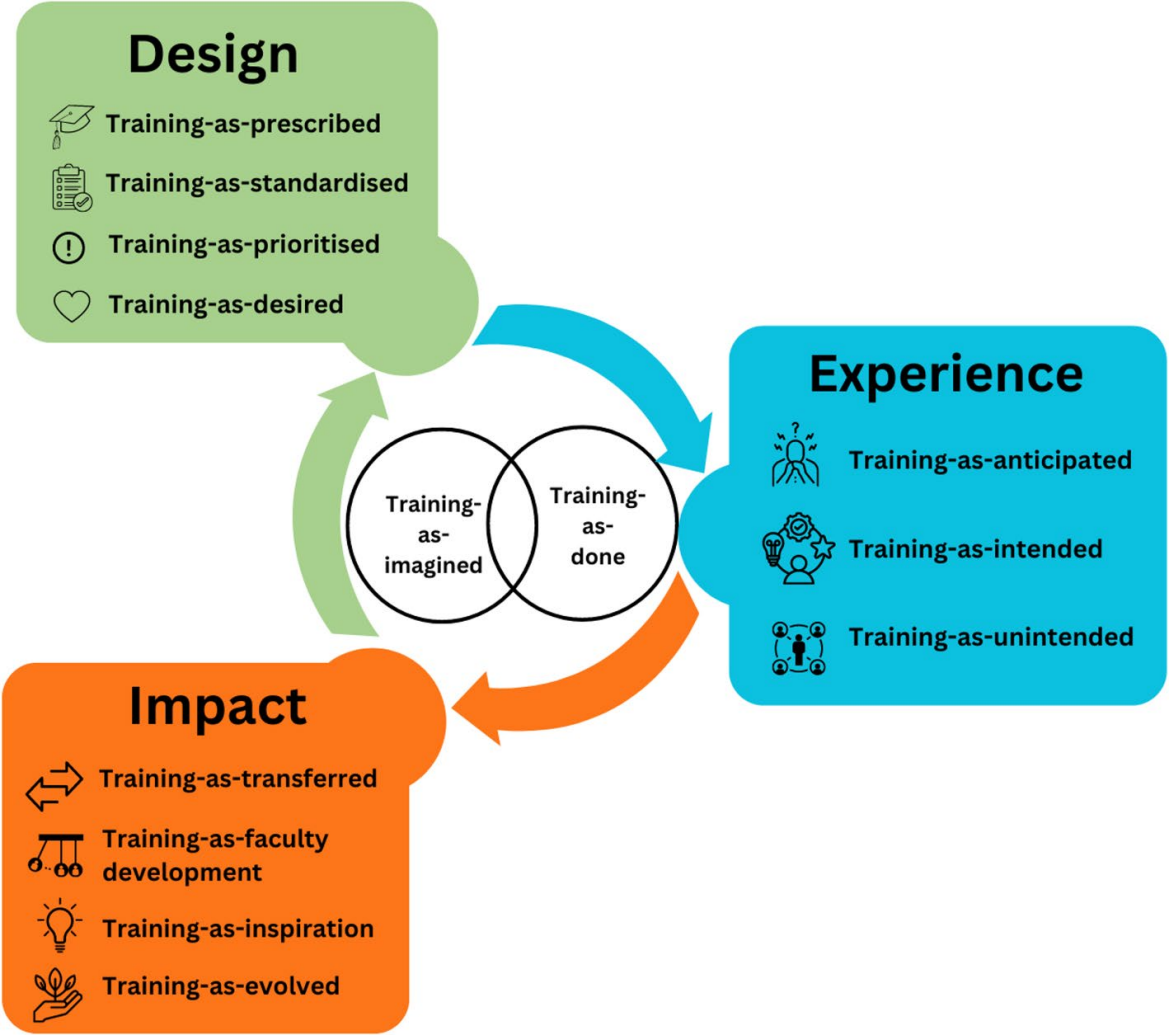
Proxies for training programme evaluation

Overall, the three domains of design, experience and impact incorporate a number of proxies for training that uncover the intended and unintended consequences of the IMT simulation programme, bringing us closer to understanding ‘training-as-done’. These are displayed in Fig. 3 below with the overlapping central concepts of training-as-imagined and training-as-done.

## Discussion

This critical realist analysis study examines a complex social intervention, the IMT simulation strategy in Scotland. Through multiple perspectives, and inspired by the proxies for work-as-done in the patient safety literature [14], this study presents proxies for training evaluation across three domains of design, experience and impact. SBE as a field has been searching for scholarly ways of exploring educational impact [13], of which this study presents an example. This study offers a framework to consider SBE impact beyond intended learning outcomes for the individual with particular calls to action heralded.

Firstly, training design must be recognised as a tension, a delicate balance, in our context between prescribed requirements, trainee needs and faculty expertise. Being cognisant of the underlying mechanisms influencing design decisions is crucial to engaging in holistic evaluative and reflexive practice. If there are significant challenges to achieving this balance, it may signal that the curriculum requires revision, and this should be escalated to appropriate stakeholders. This study reiterated the need for trainee involvement in postgraduate training design, just as there have been calls for student involvement in curricular design in undergraduate training [31]. In doing so, we increase learner agency [32] and bridge gaps between educational and workplace environments to enhance the transfer of learning. Learner involvement in the design and evaluative processes is a way educators can bring alternative and valuable perspectives and understand training-as-desired particularly when considering training adaptations. As a result, we have formally embedded an annual review day with trainee and stakeholder involvement to discuss these tensions, guided by the proxies for the training design we have described.

Secondly, using the proxies for training experience has highlighted aspects of the training experience often neglected such as training-as-anticipated. The training-as-anticipated described in this study reinforces the need to ensure psychological safety, recognising that trainees are taking a risk in peer-observed performance [33]. This was a double-edged sword, with a risk of social judgement but a desired opportunity for social comparison for their professional identity development. The findings have prompted our team to reduce the surprise elements in simulation, which can add to this anticipation, by being more transparent about learning objectives [34, 35]. Training-as-unintended highlighted social cohesion as part of the experience reiterating the need for face-to-face training, providing further justification for stakeholders and funders. Just as SBE has identified latent social impacts in clinical settings [36], so too has this study identified latent social impacts through SBE implementation. Data revealed the fostering of a shared identity which adds to the literature recognising that SBE can promote professional identity transformation [37] and a sense of social cohesion [38]. Returning to our moon analogy, this study gleaned data pertaining to aspects of the training experience that are often hidden and unacknowledged, the dark side of the moon, which the critical realist stance and proxies for training evaluation have allowed us to take stock of.

Thirdly, opportunities should be taken to be explicit with learners regarding training-as-imagined, and specifically enquire about what else happened, to capture the unintended. Educators need to be open to the inevitability that training-as-imagined will be distinct from the realities of training-as-done; actively seeking out the unintended consequences (positive and negative) of training which can feedback into design through an ongoing cycle of improvement. Simulation educators might consider alternative ways of gathering insight into such hidden impacts through informal discussions or focus groups after courses, perhaps using some of the proxies described here to frame discussions. We intend to use the proxies at our review day with trainees to be explicit about intended outcomes as well as at the introduction to each course.

Training-as-imagined is important to consider when aligning evaluation to intentions, as intentions may differ depending on various stakeholder perspectives [6]. Here, we must acknowledge the individual and contextual domains of reflexivity when undertaking educational research [39]—what are our intentions? What are our preconceived ideas? By being explicit, we can improve transparency for ourselves and others [6]. Similar to the paradigm shift from Safety I to Safety II, we are experiencing a paradigm shift from outcome evaluation to programme evaluation that could offer a more holistic, honest and useful endeavour; from proving to improving [7]. There is a move toward evaluation utilisation, the concept of evaluative practices to influence thinking and action [6]. The rhetoric of focussing only on participant experience ignores the reality that stakeholder perspectives are instrumental in supporting and funding such programmes and impact beyond the individual must be considered. The various proxies for training facilitate insight into the impact beyond individual trainees, for example influencing other courses in development.

As with most successful training programmes, there are ongoing events to be improved, not simply past events to congratulate. This requires an iterative approach to improvement, with the opportunity to capture adaptation. As a simulation community, we must strive toward holistic evaluative practices, involving multiple stakeholders and situated throughout the life of a programme, not as a snapshot [4, 40]. Embedding an iterative evaluative effort can be daunting but can help move us from ‘training-as-done’ to ‘training-as-could be’, leading to adaptation and training-as-evolved.

### Strengths and limitations

This study has operationalised theory from another discipline prompting a perspective shift in the approach to programme evaluation. By employing critical realist analysis, we can examine the particular social conditions under which causal mechanisms take effect in certain contexts [20]. Although extensive data was gathered longitudinally through questionnaire data, this interview study represents reflections after completing all three courses rather than real-time data. This was intentional to attempt to encourage trainees to provide honest reflections at a stage where they were completing their IMT training and to reduce the tendency toward favourable evaluation at the time [41]. However, a truly longitudinal approach, perhaps with audio-diaries, could have afforded different insights particularly relating to training anticipation and experience [42]. Whilst we recognise that this data represents findings in our particular context, we hope that through the use of critical realist analysis, we have identified underlying mechanisms which may be applicable and worthy of consideration in other contexts. It is argued that critical realism offers greater generalisability than many other paradigms through its potential to explain complex interventions [18].

## Conclusions

This critical realist analysis study sheds light on design tensions and latent social impacts in the postgraduate SBE context of the IMT simulation strategy in Scotland. It conceptualises a training cycle with various proxies of training evaluation, inspired by Shorrock’s varieties of human work in patient safety [14], shifting focus away from intended outcomes to a more holistic and authentic evaluative practice.

## Data Availability

Data are available from the corresponding author on reasonable request.

## Abbreviations

SBE: Simulation-based education
IMT: Internal medicine training

## References

[1] Ker J, Bradley P. Simulation in medical education. In: Swanwick T, editor. Understanding Medical Education. Wiley-Blackwell; 2007. p. 164–80.

[2] Varpio L, Sherbino J (2024). Demonstrating causality, bestowing honours, and contributing to the arms race: threats to the sustainability of HPE research. Med Educ..

[3] Kaba A, Cronin T, Tavares W, Horsley T, Grant VJ, Dube M. Improving team effectiveness using a program evaluation logic model: case study of the largest provincial simulation program in Canada. Int J Healthc Simul. 2023;1–8.

[4] Haji F, Morin MP, Parker K (2013). Rethinking programme evaluation in health professions education: beyond “did it work?”. Med Educ.

[5] Fawns T, Aitken G, Jones D (2021). Ecological teaching evaluation vs the datafication of quality: understanding education with, and around, data. Postdigital Sci Educ.

[6] Onyura B (2020). Useful to whom? Evaluation utilisation theory and boundaries for programme evaluation scope. Med Educ.

[7] Onyura B, Fisher AJ, Wu Q, Rajkumar S, Chapagain S, Nassuna J (2022). To prove or improve? Examining how paradoxical tensions shape evaluation practices in accreditation contexts. Med Educ.

[8] Nevo D (1983). The conceptualization of educational evaluation : an analytical review of the literature author. Rev Educ Res.

[9] Hosseini S, Allen L, Khalid F, Li D, Stellrecht E, Howard M (2023). Evaluation of continuing professional development for physicians – time for change: a scoping review. Perspect Med Educ.

[10] Allen LM, Hay M, Palermo C (2022). Evaluation in health professions education—is measuring outcomes enough?. Med Educ.

[11] Roberts C, Khanna P, Lane AS, Reimann P, Schuwirth L. Exploring complexities in the reform of assessment practice: a critical realist perspective. Adv Heal Sci Educ [Internet]. 2021;26(5):1641–57. Available from: 10.1007/s10459-021-10065-8 10.1007/s10459-021-10065-8 34431028

[12] LeBlanc VR. The relationship between emotions and learning in simulation-based education. Simul Healthc J Soc Simul Healthc [Internet]. 2019;14(3):137–9. Available from: http://insights.ovid.com/crossref?an=01266021-201906000-00001 10.1097/SIH.0000000000000379 31136421

[13] Hosseini S, Yilmaz Y, Shah K, Gottlieb M, Stehman CR, Hall AK (2022). Program evaluation: an educator’s portal into academic scholarship. AEM Educ Train.

[14] Shorrock S. The varieties of human work. Saf Differ. 2017;1–11. Available from: http://www.safetydifferently.com/the-varieties-of-human-work/

[15] Hollnagel E (2015). From safety-I to safety-II : a white paper.

[16] Maxwell JA (2012). A realist approach for qualitative research.

[17] Moon K, Blackman D (2014). A guide to understanding social science research for natural scientists. Conserv Biol.

[18] Ellaway RH, Kehoe A, Illing J (2020). Critical realism and realist inquiry in medical education. Acad Med.

[19] Wong G, Greenhalgh T, Westhorp G, Pawson R (2012). Realist methods in medical education research: what are they and what can they contribute?. Med Educ.

[20] Fletcher AJ. Applying critical realism in qualitative research: methodology meets method. Int J Soc Res Methodol. 2017;20(2):181–94. Available from: 10.1080/13645579.2016.1144401

[21] Gorski PS (2013). What is critical realism? And why should you care?. Contemp Sociol A J Rev.

[22] Lawani A (2020). Critical realism: what you should know and how to apply it. Qual Res J.

[23] Cheng A, Kessler D, Mackinnon R, Chang TP, Nadkarni VM, Hunt EA (2016). Reporting guidelines for health care simulation research: extensions to the CONSORT and STROBE statements. Adv Simul.

[24] Roberts C, Khanna P, Bleasel J, Lane S, Burgess A, Charles K (2022). Student perspectives on programmatic assessment in a large medical programme: a critical realist analysis. Med Educ.

[25] Thistlethwaite JE (2015). When I say … realism. Med Educ.

[26] Bhaskar R (1979). The possibility of naturalism: a philosophical critique of the natural sciences.

[27] The Joint Royal College of Physicians Training Board. Curriculum for internal medicine stage 1 training- implementation. August 2019. 2017. https://www.thefederation.uk/training/training-certification/training-pathways. Accessed 25 June 2024.

[28] Quraishi S, Wade W, Black D (2019). Development of a GMC aligned curriculum for internal medicine including a qualitative study of the acceptability of ‘capabilities in practice’ as a curriculum model. Futur Healthc J.

[29] Scahill EL, Oliver NG, Tallentire VR, Edgar S, Tiernan JF, Scahill EL (2021). An enhanced approach to simulation-based mastery learning optimising the educational impact of a novel national postgraduate medical boot camp. Adv Simul.

[30] Olsen W (2007). Critical realist explorations in methodology. Methodol Innov Online.

[31] McLean M, Gibbs T (2010). Twelve tips to designing and implementing a learner-centred curriculum: prevention is better than cure. Med Teach.

[32] Watling C, Ginsburg S, LaDonna K, Lingard L, Field E (2021). Going against the grain: an exploration of agency in medical learning. Med Educ.

[33] Purdy E, Borchert L, El-Bitar A, Isaacson W, Bills L, Brazil V (2022). Taking simulation out of its “safe container”—exploring the bidirectional impacts of psychological safety and simulation in an emergency department. Adv Simul.

[34] Monteiro S, Sibbald M (2020). Aha! Taking on the myth that simulation-derived surprise enhances learning. Med Educ.

[35] Somerville SG, Harrison NM, Lewis SA. Twelve tips for the pre-brief to promote psychological safety in simulation-based education. Med Teach. 2023;45(12):1349–56. Available from: 10.1080/0142159X.2023.2214305 10.1080/0142159X.2023.2214305 37210674

[36] Brydges R, Nemoy L, Ng S, Khodadoust N, Léger C, Sampson K (2021). Getting everyone to the table: exploring everyday and everynight work to consider ‘latent social threats’ through interprofessional tabletop simulation. Adv Simul.

[37] Kainth R, Reedy G. Transforming professional identity in simulation debriefing. Simul Healthc J Soc Simul Healthc. 2023;Publish Ah(00):1–15. 10.1097/SIH.0000000000000734 37335122

[38] Smith SE, Tallentire VR. Simulation for social integration. Int J Healthc Simul. 2023;1–9.

[39] Olmos-Vega FM, Stalmeijer RE, Varpio L, Kahlke R. A practical guide to reflexivity in qualitative research: AMEE Guide No. 149. Med Teach [Internet]. 2023;45(3):241–51. Available from: 10.1080/0142159X.2022.2057287 10.1080/0142159X.2022.2057287 35389310

[40] Hamza DM, Regehr G (2021). Eco-Normalization: evaluating the Longevity of an Innovation in Context. Acad Med.

[41] Scriven M (2010). Evaluation bias and its control*. J Multidiscip Eval.

[42] Gordon L (2021). Making space for relational reflexivity in longitudinal qualitative research. Med Educ.

